# Supplementary material for: Content-rich biological network constructed by mining PubMed abstracts
Source: BMC Bioinformatics. 2004 Oct 8;5:147. doi: 10.1186/1471-2105-5-147 (PMC528731; doi:10.1186/1471-2105-5-147)
Supplement: Additional File 2 — The original results of the above study (non-essential files are deleted to keep the file size under the limit set by BMC bioinformatics). [file 1471-2105-5-147-S2.bz2 › chilibotAdditionalFile2/dip50/496ID10187774E10659/html/ACVR2_ACVRL1.html]

 


 **ACVR2** and **ACVRL1** 
  
Found 62 abstracts in PubMed, retrieved 50.  
 

 What does Google say? 
 PDF only 
| .edu only 

---

**Interactive relationship** (e.g. stimulation, inhibition, etc)

**Neutral relationship**- BMP 4 and GDF 5 are known to bind to activin receptor  [ **ACVR2** ]  like kinase 3 ALK  [ **ACVRL1** ]  3 and or ALK  [ **ACVRL1** ]  6 also termed BMP type IA and type IB receptors, respectively.  Ref: 11282024 J Cell Sci, 2001
- BMP 7 OP 1 was found to bind Activin receptor  [ **ACVR2** ]  like kinase ALK 1  [ **ACVRL1** ]  as well as ALK  [ **ACVRL1** ]  3 BMPR IA in ATDC5 cells.  Ref: 9125848 Kokubyo Gakkai Zasshi, 1997
- Activin receptor  [ **ACVR2** ]  like kinase 1 ALK 1  [ **ACVRL1** ] , the gene mutated in HHT  [ **ACVRL1** ]  type 2 HHT2  [ **ACVRL1** ] , is a serine threonine kinase receptor type I of the TGF beta superfamily, specifically expressed on endothelial cells.  Ref: 12700602 Eur J Hum Genet, 2003
- HHT  [ **ACVRL1** ]  type 2 is caused by loss of function mutations in activin receptor  [ **ACVR2** ]  like kinase 1  **ACVRL1**  or ALK1  [ **ACVRL1** ] .  Ref: 12588795 Hum Mol Genet, 2003
- This strongly suggests that B1 ALK  [ **ACVRL1** ]  4 ActR IB could be the type I activin receptor  [ **ACVR2** ] , as type I and type II activin receptor  [ **ACVR2** ]  were supposed to form a receptor complex.  Ref: 9013782 Brain Res Mol Brain Res, 1996
- Genetic studies have demonstrated that HHT  [ **ACVRL1** ]  can be caused by loss of function mutations in the gene encoding activin receptor  [ **ACVR2** ]  like kinase 1  **ACVRL1** .  Ref: 11062473 Nat Genet, 2000
- Furthermore, the effects of ALK  [ **ACVRL1** ]  2\* signalling are at odds with it behaving as an activin receptor  [ **ACVR2** ]  in the early Xenopus embryo.  Ref: 9367435 Development, 1997
- Hereditary hemorrhagic telangiectasia HHT  [ **ACVRL1** ]  is a vascular disorder in humans which has been mapped to two genes, endoglin and activin receptor  [ **ACVR2** ]  like kinase 1 ALK 1  [ **ACVRL1** ]  both of which mediate signaling by transforming growth factor beta ligands in vascular endothelial cells.  Ref: 12667943 Cardiovasc Res, 2003
- ALK 1  [ **ACVRL1** ]  activin receptor  [ **ACVR2** ]  like kinase 1, a type I receptor of the transforming growth factor TGF beta superfamily, is the gene mutated in hereditary hemorrhagic telangiectasia type 2 HHT2  [ **ACVRL1** ]  while endoglin is mutated in HHT1.  Ref: 10767348 Hum Mol Genet, 2000

**Non-interactive relationship** (e.g. studied together, co-existance, homology, etc.)

- Activin A bound to ActR IB ALK  [ **ACVRL1** ]  4 and ActR II  [ **ACVR2** ] , and BMP 7 bound to ActR I ALK  [ **ACVRL1** ]  2, BMP type I receptor BMPR I ALK  [ **ACVRL1** ]  3, ActR II  [ **ACVR2** ]  and BMPR II.  Ref: 10574705 J Cell Sci, 1999
- BMP 6 strongly bound to activin receptor  [ **ACVR2** ]  like kinase ALK  [ **ACVRL1** ]  2 also termed ActR I, together with type II receptors, i.e. BMP type II receptor BMPR II and activin type II receptor ActR II  [ **ACVR2** ] .  Ref: 10504300 J Cell Sci, 1999
- In the two largest families, we used microsatellite markers to test for linkage to genes encoding TGF beta receptor proteins, including endoglin and activin receptor  [ **ACVR2** ]  like kinase 1 ALK1  [ **ACVRL1** ] , and BMPR2.  Ref: 11484689 N Engl J Med, 2001
- Recent studies have identified mutations of the bone morphogenetic protein receptor 2 BMPR2 gene and the activin receptor  [ **ACVR2** ]  like kinase 1 ALK1  [ **ACVRL1** ]  gene, that affect the transforming growth factor beta TGF beta receptor superfamily, a group of transmembrane signaling molecules with serine threonine kinase activity that are involved in the regulation of cell growth.  Ref: 12624796 Z Rheumatol, 2003
- The species specific sequence information for the two T beta R I, namely, activin receptor  [ **ACVR2** ]  like kinase 5 ALK  [ **ACVRL1** ]  5 and Tsk7L, in the rat was sought.  Ref: 9321911 Am J Physiol, 1997
- A mechanistic explanation for increased pSmad1 5 staining during inflammation was provided by the upregulated expression of all the BMP type I receptors, i.e., activin receptor  [ **ACVR2** ]  like kinase ALK  [ **ACVRL1** ]  2, ALK3, and ALK6, in the inflamed bronchial epithelial cells.  Ref: 12151307 Am J Respir Cell Mol Biol, 2002
- The type I receptors examined were the TGF beta type I receptor TbetaR I, activin type I receptor ActR I, activin type IB receptor ActR IB, bone BMP type IA receptor BMPR IA, BMP type IB receptor BMPR IB, and activin receptor  [ **ACVR2** ]  like kinase 1 ALK 1  [ **ACVRL1** ] .  Ref: 10321529 Acta Ophthalmol Scand, 1999
- To explore the molecular cascade of odontoblast differentiation, Northern blot analyses and reverse transcriptase polymerase chain reaction were here used to investigate the expression patterns of the genes for TGF beta superfamily members TGF beta 1, namely bone morphogenetic protein BMP 4, BMP 7, activin beta A and activin beta B, and their type I and type II receptors, namely activin receptor  [ **ACVR2** ]  like kinase ALK  [ **ACVRL1** ]  2 ActR I, ALK  [ **ACVRL1** ]  3 BMPR IA, ALK  [ **ACVRL1** ]  4 ActR IB, ALK  [ **ACVRL1** ]  5 T beta R I, BMPR II and T beta R II, during differentiation of pulp cells into preodontoblasts in bovine adult pulp cell culture.  Ref: 9296267 Arch Oral Biol, 1997
- To understand the roles of TGF beta isoforms TGF beta and 3 and TGF beta type I receptors activin receptor  [ **ACVR2** ]  like kinase ALK  [ **ACVRL1** ] , 5 and 6 in PDL cell differentiation, their expression was investigated using Northern blot analysis.  Ref: 10633885 J Periodontal Res, 1999
- Here we report the isolation and characterization of a novel type I RSTK termed activin receptor  [ **ACVR2** ]  like kinase 7 ALK  [ **ACVRL1** ]  7 that, unlike other members of this receptor family, is predominantly expressed in the adult central nervous system.  Ref: 8940033 J Biol Chem, 1996
- Moreover, GDF 5 transduced the signal efficiently by ALK  [ **ACVRL1** ]  6 BMPR IB in the presence of BMPR II or ActR II  [ **ACVR2** ] .  Ref: 9125848 Kokubyo Gakkai Zasshi, 1997
- To investigate the expression and function of specific Type I TGFbeta receptors during AV cushion transformation, we cloned and characterized the chicken homologues of two mammalian activin receptor  [ **ACVR2** ]  like kinases ALK  [ **ACVRL1** ] , ALK2 and ALK5, and generated specific, polyclonal antibodies against the extracellular binding domains of each.  Ref: 10885742 Dev Biol, 2000
- In Xenopus embryo, Smad8 is able to transcriptionally activate a subset of mesoderm target genes similar to those induced by the receptor serine kinase, activin receptor  [ **ACVR2** ]  like kinase ALK  [ **ACVRL1** ]  2.  Ref: 9371779 Proc Natl Acad Sci U S A, 1997
- The expression of both type I ALK  [ **ACVRL1** ]  4 ActRIB and type II ActRII  [ **ACVR2** ]  receptors for activin was detected in F5 5.fl cells.  Ref: 11078996 Eur J Endocrinol, 2000
- Two related genes, endoglin and activin receptor  [ **ACVR2** ]  like kinase ALK 1  [ **ACVRL1** ] , have been mapped to chromosomes 9q34 and 12q13, respectively.  Ref: 11802521 J Formos Med Assoc, 2001
- In man, these include the activin receptor  [ **ACVR2** ]  like kinase 3 ALK  [ **ACVRL1** ]  3, a type I receptor protein.  Ref: 8707881 J Cell Physiol, 1996
- Here, we characterize a small molecule inhibitor SB 431542 that was identified as an inhibitor of activin receptor  [ **ACVR2** ]  like kinase ALK  [ **ACVRL1** ]  5 the TGF beta type I receptor.  Ref: 12065756 Mol Pharmacol, 2002
- Most cases are caused by mutations in the endoglin gene on chromosome 9 HHT  [ **ACVRL1** ]  type 1 or the activin receptor  [ **ACVR2** ]  like kinase 1 gene on chromosome 12 HHT  [ **ACVRL1** ]  type 2, which leads to telangiectases and arteriovenous malformations AVM of the skin, mucosa, and viscera.  Ref: 11773580 Pediatrics, 2002
- Activin receptor  [ **ACVR2** ]  like kinase 5 ALK  [ **ACVRL1** ]  5 is a TGF beta type I receptor expressed in various TGF beta responsive cells.  Ref: 12384983 J Cell Physiol, 2002
- BMPR I group and those of activin receptor  [ **ACVR2** ]  like kinase ALK 1  [ **ACVRL1** ]  and ALK  [ **ACVRL1** ]  2 ALK 1  [ **ACVRL1** ]  group induced alkaline phosphatase activity in C2C12 cells.  Ref: 10564272 Mol Biol Cell, 1999
- We show that vbg encodes activin receptor  [ **ACVR2** ]  like kinase 1  **Acvrl1** .  Ref: 12050147 Development, 2002
- One of the causative genes is the activin receptor  [ **ACVR2** ]  like kinase 1 ALK 1  [ **ACVRL1** ]  gene located on chromosome 12q13.  Ref: 10694922 Hum Mutat, 1998
- Activin receptor  [ **ACVR2** ]  like kinase 1 ALK 1  [ **ACVRL1** ]  has homology to the type I receptor family, but its ligand and corresponding type II receptor are unknown.  Ref: 10187774 J Biol Chem, 1999
- Mutant D27K has a 2 fold higher specific bio activity and binding affinity to an ActRIIA  [ **ACVR2** ]  ALK  [ **ACVRL1** ]  4 activin receptor  [ **ACVR2** ]  complex than wild type activin.  Ref: 10092672 J Biol Chem, 1999
- We thus demonstrate here that, while TbetaRI is crucial for the function of TGF beta during vascular development and can not be compensated for by the activin receptor  [ **ACVR2** ]  like kinase 1 ALK 1  [ **ACVRL1** ] , functional hematopoiesis and development of hematopoietic progenitors is not dependent on TGF beta signaling via TbetaRI.  Ref: 11285230 EMBO J, 2001
- This was accompanied by strong upregulation of the activin receptor  [ **ACVR2** ]  ALK  [ **ACVRL1** ]  4 ActR IB and redistribution of the TGF beta responsive ALK  [ **ACVRL1** ]  5 TbetaR I.  Ref: 11472976 Am J Respir Cell Mol Biol, 2001
- In COS 1 transfected cells, ALK 1  [ **ACVRL1** ]  was found in the TGF beta1 and beta3 receptor complexes in association with endoglin and TbetaRII, but not in activin receptor  [ **ACVR2** ]  complexes containing endoglin.  Ref: 10767348 Hum Mol Genet, 2000
- Surprisingly, activin had no effect on follistatin mRNA levels, despite the fact that FS D1h cells express ActRII  [ **ACVR2** ] , ActRIIB, and ALK  [ **ACVRL1** ]  4 ActRIB.  Ref: 12538636 Endocrinology, 2003
- This study examines the osteogenic potential of CDMP 1, CDMP 2, BMP 6 and osteogenic protein 1 OP 1 in bone marrow stromal cells BMSC and investigates the endogenous expression of CDMPs BMPs and their respective activin receptor  [ **ACVR2** ]  like kinase ALK  [ **ACVRL1** ]  receptors.  Ref: 11052813 Cytokine, 2000
- The localizations of serine threonine kinase receptor mRNA for the novel type I TGF beta and or activin receptor  [ **ACVR2** ]  named B1 rat, ALK  [ **ACVRL1** ]  4 mouse or ActR IB human were demonstrated by in situ hybridization.  Ref: 9013782 Brain Res Mol Brain Res, 1996
- Constitutively active forms of BMP type I receptors BMPR IA and BMPR IB and those of activin receptor  [ **ACVR2** ]  like kinase ALK 1  [ **ACVRL1** ]  and ALK  [ **ACVRL1** ]  2 maintained differentiation of chondrocytes in the presence of retinoic acid.  Ref: 12565908 Biochem Biophys Res Commun, 2003
- We then investigated whether the sensitivity of the early long bone rudiments to OP 1 correlated with high expression of the OP 1 binding type I serine threonine kinase receptors activin receptor  [ **ACVR2** ]  like kinase ALK  [ **ACVRL1** ]  2 ActR I, ALK  [ **ACVRL1** ]  3 BMPR IA or ALK  [ **ACVRL1** ]  6 BMPR IB at this early stage.  Ref: 10705576 Growth Factors, 2000
- The activin receptor  [ **ACVR2** ]  like kinase 1 ALK1  [ **ACVRL1** ]  is a type I receptor for transforming growth factor beta TGF beta family proteins.  Ref: 10716993 Proc Natl Acad Sci U S A, 2000
- Activin receptor  [ **ACVR2** ]  like kinase ALK 1  [ **ACVRL1** ] , ALK  [ **ACVRL1** ]  2, ALK  [ **ACVRL1** ]  3, and ALK  [ **ACVRL1** ]  6 were able to reproduce the hypertrophic maturation of chondrocytes induced by BMP 2.  Ref: 12082094 J Biol Chem, 2002
- We expressed the kinase domain of the TGF beta type I receptor activin receptor  [ **ACVR2** ]  like kinase ALK  [ **ACVRL1** ]  5 and the substrate, Smad3, and determined that SB 431542 is a selective inhibitor of Smad3 phosphorylation with an IC50 of 94 nM.  Ref: 12065755 Mol Pharmacol, 2002
- A preliminary investigation was performed to exclude association between the predisposition and certain candidate genes including KDR kinase insert domain receptor, TEK TEK tyrosine kinase endothelial,  **ACVRL1**  activin receptor  [ **ACVR2** ]  like kinase 1, ENG endoglin and FLT4 fms like tyrosine kinase 4.  Ref: 12752572 Clin Genet, 2003
- SB 431542 is a potent and specific inhibitor of transforming growth factor beta superfamily type I activin receptor  [ **ACVR2** ]  like kinase ALK  [ **ACVRL1** ]  receptors ALK4, ALK5, and ALK7.  Ref: 12065756 Mol Pharmacol, 2002
- Reverse transcription polymerase chain reaction of PASMC mRNA detected transcripts for type I TGF betaRI, Alk 1  [ **ACVRL1** ] , ActRI, and BMPRIB and type II TGF betaRII, BMPR II, ActRII  [ **ACVR2** ] , ActRIIB receptors.  Ref: 11502704 Circulation, 2001
